# Cottonseed Meal Protein Isolate as a New Source of Alternative Proteins: A Proteomics Perspective

**DOI:** 10.3390/ijms231710105

**Published:** 2022-09-03

**Authors:** Chee Fan Tan, Soon Hong Kwan, Chun Shing Lee, Yan Ni Annie Soh, Ying Swan Ho, Xuezhi Bi

**Affiliations:** 1 Bioprocessing Technology Institute (BTI), Agency for Science, Technology and Research (A*STAR), Singapore 138668, Singapore; 2 Duke-NUS Medical School, National University of Singapore, Singapore 169857, Singapore

**Keywords:** cottonseed meal, gossypol, proteomics, mass spectrometry, food, alternative protein, digestibility, allergen analysis, taste profile

## Abstract

Cottonseed meal (CSM) is a good source of dietary proteins but is unsuitable for human consumption due to its gossypol content. To unlock its potential, we developed a protein extraction process with a gossypol removal treatment to generate CSM protein isolate (CSMPI) with ultra-low gossypol content. This process successfully reduced the free and total gossypol content to 4.8 ppm and 147.2 ppm, respectively, far below the US FDA limit. In addition, the functional characterisation of CSMPI revealed a better oil absorption capacity and water solubility than pea protein isolate. Proteome profiling showed that the treatment improved protein identification, while SDS-PAGE analysis indicated that the treatment did not induce protein degradation. Amino acid analysis revealed that post-treated CSMPI was rich in branched-chain amino acids (BCAAs). Mass spectrometry analysis of various protein fractions obtained from an in vitro digestibility assay helped to establish the digestibility profile of CSM proteins. Several potential allergens in CSMPI were also found using allergenic prediction software, but further evaluation based on their digestibility profiles and literature reviews suggests that the likelihood of CSMPI allergenicity remains low. Overall, our results help to navigate and direct the application of CSMPIs as alternative proteins toward nutritive human food application.

## 1. Introduction

Cottonseed meal (CSM) is an agro-by-product derived from cottonseed after oil extraction. Fifteen million tons of CSM were estimated to be produced annually between 2009 and 2018 [1]. CSM has a high protein content ranging from 30–50%, depending on the dehulling process [2]. The high amount of proteins within makes CSM an excellent source of high-quality protein ingredients to meet the global protein demand for livestock and human consumption. However, most CSM animal feeds are mainly suitable for adult ruminants, as a report has indicated that adult ruminants can tolerate the toxic gossypol compound found in cottonseed [3]. Gossypol is a polyphenolic compound found predominantly in the cottonseed pigment glands. It exists as a free form or binds to lysine and/or arginine during heating [4]. The consumption of a high amount of free gossypol can cause acute toxicity, leading to respiratory distress, weakness and even fatality in animals [5]. At the same time, the human consumption of cottonseed oil that contains gossypol has also been associated with infertility [6]. On the other hand, bound gossypol is considered nontoxic, but it reduces the bioavailability of the amino acid that it is bound to. Furthermore, some reports have shown that bound gossypol might be released from the proteins during digestion [7,8].

Gossypol content in CSM varies depending on the oil extraction process, and the concentration can range between 200 and 177,000 parts per million (ppm) for free gossypol and between 9300 and 14,300 ppm for total gossypol [9]. The United States Food and Drug Administration (FDA) mandated that the free gossypol concentration in a food product must be less than 450 ppm to ensure that it is safe for human consumption (US FDA, 21CFR73.140 and 21CFR172.894). Hence, researchers and commercial partners have explored the possibilities of developing glandless cottonseed to eliminate gossypol [10,11]. However, glandless cotton plants are highly susceptible to pests, severely affecting their harvest. The current advancement in genetic modification technology has created a genetically modified cotton plant, TAM66274, with targeted reduction in gossypol content in the cottonseed only. This ultra-low gossypol cottonseed (free gossypol: 300 ppm) is the first cottonseed product approved by FDA for human consumption [12]. Nonetheless, genetically modified products face regulatory issues in certain countries [13] and remain poorly received by the public [14,15].

Other gossypol removal methodologies are also being developed, including microbial fermentation [16,17], and aqueous- and/or solvent-based extraction [18,19,20,21]. Zhang et al. demonstrated the ability of rumen *Bacillus subtilis* to reduce free and total gossypol content in CSM with higher essential amino acid content through fermentation [16]. Although the free gossypol content was successfully reduced from 289.8 ppm to 61.25 ppm after microbial fermentation, this process is time-consuming (~72 h), and the scalability issue remains unresolved. Aqueous-/solvent-based cottonseed protein extraction with gossypol removal remains an important area of research as the process is faster and has better scalability in producing cottonseed meal protein with low gossypol content than the fermentation methodology. Gossypol is highly soluble in the organic solvent, and ethanol usage is favoured over acetone due to its fast gossypol-removing capacity [19]. Ethanol is also more environmentally friendly than acetone. Furthermore, Pelitire et al. reported that ethanol acidified with tribasic acids, such as phosphoric acid, released bound gossypol at an elevated temperature, reducing the total gossypol content from 11,700 ppm to 640 ppm [19]. On the other hand, Kumar et al. developed an aqueous-based process to produce an ultra-low-gossypol cottonseed protein with free and total gossypol levels of 29 ppm and 270 ppm, respectively [20]. However, this method requires a high-speed centrifuge that it may not be feasible to scale up in an industrial setting.

This study aims to elucidate the proteins extracted from cottonseed meal for food application, possibly as a new source of alternative proteins. An ultra-low-gossypol cottonseed meal protein isolate (CSMPI) was generated using our developed protein extraction method. This process was based on the principle of an alkaline-based protein extraction methodology, followed by a multistep acidified organic-solvent-based treatment to remove both free and bound gossypol. The proteomes of the pre-treated and post-treated protein isolates were profiled using advanced mass spectrometry to study the effect of gossypol removal treatment on CSMPI. In addition, various functionality tests were conducted to compare the functional attributes of post-treated CSMPI with pea protein isolate. Subsequently, the potential food allergens and taste profile of the CSMPI were also evaluated through in silico protein sequence analysis. Lastly, post-treated CSMPI was subject to an in vitro digestibility assay and both protease-sensitive and -resistant protein fractions were obtained for LC-MS/MS analysis to evaluate the digestibility profile of CSMPI.

## 2. Results

### 2.1. Overview of the Protein Extraction and Gossypol Removal Workflow to Produce Ultra-Low Gossypol CSMPI

**Figure 1:**
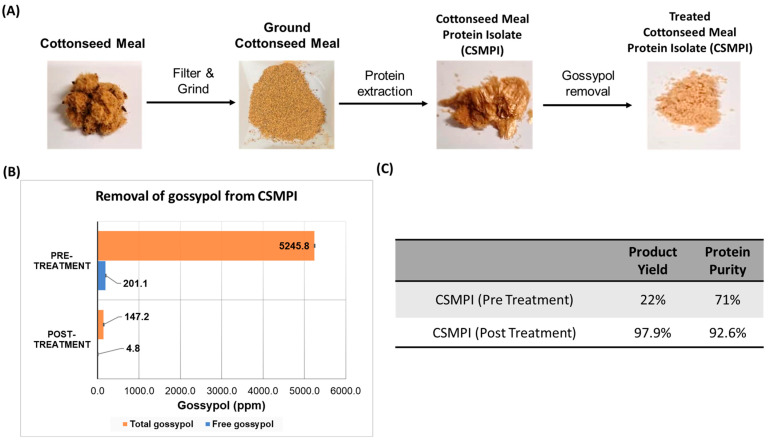
An overview of the CSMPI production process. (**A**) The general workflow of the protein extraction process to obtain CSMPI with ultra-low gossypol content from raw CSM. (**B**) Comparison of free and total gossypol between the pre-treated and post-treated CSMPI. (**C**) The product yield and protein purity of the pre-treated CSMPI and post-treated CSMPI.

As the cottonseed meal still contains a significant amount of cotton fibre and other impurities, these must be removed before the protein isolate extraction. As shown in Figure 1A, the CSM was first ground and filtered using a sieve to remove all the remaining fibre content. The rest of the workflow illustrates the extraction and treatment processes to generate CSMPI with ultra-low gossypol content from the raw CSM using a modified solvent-extraction method. The end product was in powder form and beige in colour. Figure 1C shows that pre-treated CSMPI’s yield was at 22% of the raw material, with a protein purity of 71%. Interestingly, the purity of the protein isolate was subsequently improved to 92.6% after the gossypol removal treatment, where most of the gossypol was removed. Thus, the gossypol removal step did not result in significant material loss as the post-treatment yield was still at 97.9%.

Figure 1B compares the gossypol amount before and after the gossypol removal treatment. The pre-treated CSMPI had high amounts of free gossypol (FG) and total gossypol (TG). Through a multistep acidified ethanol treatment, HPLC analysis of the FG and TG content from post-treated CSMPI revealed FG reduction from 201.1 ppm to 4.8 ppm and TG reduction from 5245.8 ppm to 147.2 ppm. The FG concentration was obtained before introducing the complexing agent to measure TG.

A good understanding of the functional properties of our post-treated CSMPI is essential for food industry adoption as these properties will guide their application, processing and storage [22]. Therefore, we evaluated several functional properties of CSMPI, including its water absorption capacity (WAC), oil absorption capacity (OAC), water solubility, emulsion properties (emulsifying activity index (EAI) and emulsion stability index (ESI)) and foaming properties (foam capacity (FC) and foam stability (FS)), and compared these properties with pea protein isolate (PPI), as shown in Table 1. CSMPI had superior OAC and water solubility but poorer WAC and EAI against PPI. On the other hand, there was no significant difference in the emulsion stability generated by both protein isolates. Lastly, both protein isolates had similar foaming properties (Table 1, Figure S2).

### 2.2. Proteomics Analysis Identified More Proteins in CSMPI after Gossypol Removal Treatment

**Figure 2:**
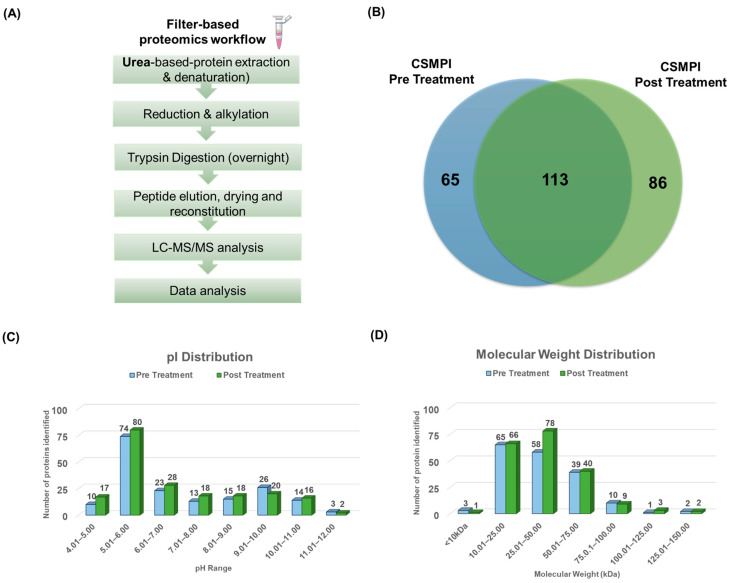
LC-MS/MS analysis of pre-treated and post-treated CSMPI. (**A**) A filter-based proteomics workflow was applied to generate tryptic peptides from CSMPI for LC-MS/MS analysis. (**B**) Venn diagram illustrating the number of common and unique proteins identified in each CSMPI dataset. (**C**) pI distribution of identified proteins from pre-treated and post-treated CSMPI. (**D**) Molecular-weight distribution of identified proteins from pre-treated and post-treated CSMPI.

**Figure 3:**
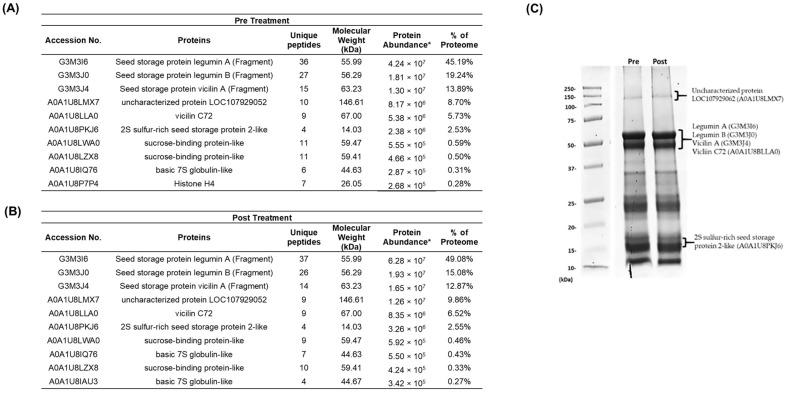
Proteomics analysis of the most abundant proteins in CSMPI. (**A**) Top 10 most abundant proteins in pre-treated CSMPI. (**B**) Top 10 most abundant proteins in post-treated CSMPI. (**C**) SDS-PAGE image of the pre-treated and post-treated CSMPI on a 12% polyacrylamide gel. * Protein Abundance is based on peptide feature area.

Label-free LC-MS/MS quantitative analysis was conducted to evaluate the quality of the CSMPI proteome after gossypol removal treatment. Firstly, tryptic peptides were obtained from CSMPI using a filter-based proteomics workflow, as shown in Figure 2A. Then, the samples were analysed using a high-resolution SCIEX TripleTOF^®^ 6600+ system for protein identification. The data files were generated from three biological replicates for each condition and processed using PEAKS^®^ Studio XPro. The correlation coefficients between the three biological replicates of pre-treated CSMPI were between 0.9039 and 0.9401 (Figure S1A), and between 0.9474 and 0.9601 for the post-treated CSMPI samples (Figure S1B). Based on the filtering criteria of at least two unique peptides and a peptide false-discovery rate (FDR) at <1%, 178 and 199 proteins were identified in pre-treated CSMPI and post-treated CSMPI, respectively. Of all the proteins identified, 113 were found in both conditions, while 65 were unique to pre-treated CSMPI and 86 were unique to post-treated CSMPI (Figure 2B). Next, the pI and molecular-weight (MW) distribution of the proteins identified were evaluated to understand the effect of the gossypol removal treatment on the proteome. The pI distribution of the proteins identified in both pre-treated and post-treated CSMPI were in the pH range of 4 to 12 (Figure 2C). More proteins were identified in the post-treated CSMPI, generally between the pI values of 4 and 9, contrary to the higher protein identification with a pI range of 9 to 10 found in the pre-treated CSMPI. Next, the molecular-weight distribution of the CSMPI was not overly affected by the gossypol removal treatment, with most of the identified proteins found in the 10–75 kDa range (Figure 2D), which is similar to the SDS-PAGE profile in Figure 3C. However, the gossypol removal treatment facilitated an increase in unique protein identification in the range of 25–50 kDa.

### 2.3. Gossypol Removal Treatment Does Not Affect the Protein Integrity of CSMPI

Seed storage proteins highly dominate the cottonseed meal proteome. They serve mainly as nitrogen, carbon and sulphur reserves, and are rapidly utilised during seed germination [23]. Our proteomics analysis revealed that 95.28% and 95.96% of the pre-treated and post-treated CSMPI proteome were seed storage proteins (Figure 3A,B). Generally, the composition of the major seed storage proteins was similar in both conditions, with Legumin A (G3MI6) being the most abundant protein in CSMPI. The next on the list are Legumin B (G3M3J0), vicilin A (G3M3J4), uncharacterised protein LOC107929052 (A0A1U8LMX7) (90% sequence similarity with vicilin C72 from *Gossypium australe*), vicilin C72 (A0A1U8LLA0) and 2S sulphur-rich seed storage protein 2-like (A0A1U8PKJ6). The relative abundance of these seed storage proteins was also visualised through SDS-PAGE analysis of the protein bands from pre-treated and post-treated CSMPI (Figure 3C). The most intense band was observed around 50 kDa, represented mainly by legumin and vicilin proteins. On the other hand, the SDS-PAGE analysis indicated that the gossypol removal treatment did not induce any observable protein degradation to the CSMPI as the protein bands detected in the pre-treated CSMPI were still visible in the post-treated sample.

**Figure 4:**
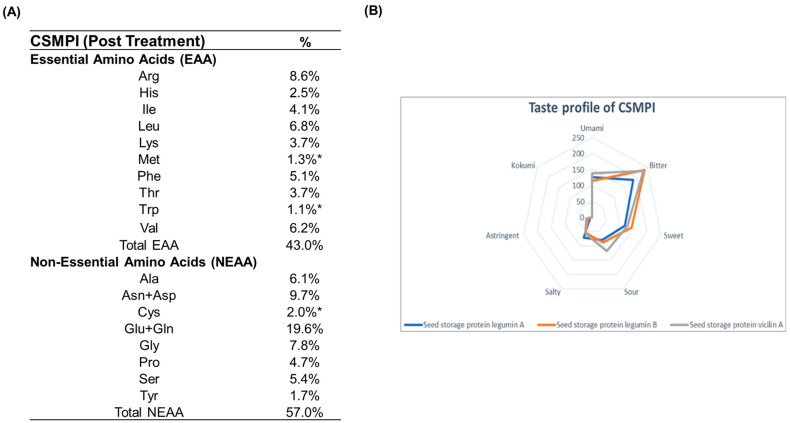
Amino acid analysis of post-treated CSMPI. (**A**) Amino acid composition of post-treated CSMPI. (**B**) Taste profile of the top 3 most abundant proteins of CSMPI. * The percentages of amino acids are calculated using a molar-ratio normalized sequence-based analysis.

The amino acid composition of post-treated CSMPI was determined through UPLC-UV analysis after acid hydrolysis. However, as acid hydrolysis results in the inaccurate determination of cysteine, methionine and tryptophan, an individual-protein normalized sequence-based analysis was performed to calculate the percentage of these three amino acids in the post-treated CSMPI. The analysis revealed the presence of all 20 amino acids in post-treated CSMPI (Figure 4A). The essential amino acids comprised 43% of the protein content and they were particularly rich in arginine, leucine, and valine. The post-treated CSMPI also had a lysine content of 3.7%. Additionally, glutamic acid/glutamine, asparagine/aspartic acid and glycine were the dominating non-essential amino acids in the post-treated CSMPI. Next, we also predicted the sensory profile of the top three most abundant proteins, the same in both the pre-treated and post-treated CSMPI, via in silico analysis. As shown in Figure 4B, these proteins had a general inclination towards a bitter taste. Sweet and umami tastes were also substantial based on the distribution, followed by a sour taste.

### 2.4. Digestibility Protein Profile and In Silico Allergenic Analysis Identified Potential Allergens in Post-Treated CSMPI Digested with Different Enzymes

**Figure 5:**
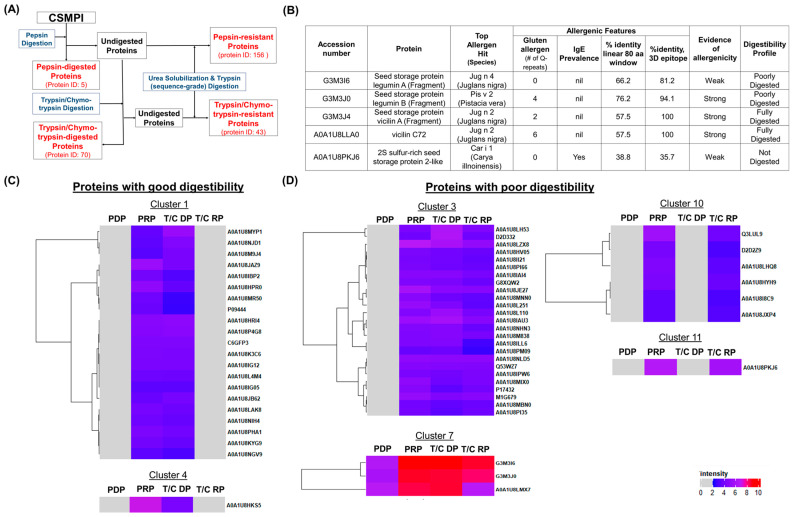
The digestibility and allergenic profile of CSMPI. (**A**) Schematic workflow of the in vitro digestibility assay with 4 protein fractions (highlighted with red text) collected for LC-MS/MS analysis. (**B**) The allergenic profile of the top 5 most abundant CSM proteins. (**C**) CSM proteins with a good digestibility profile. (**D**) CSM proteins with a poor digestibility profile. PDP: pepsin-digested protein; PRP: pepsin-resistant protein; T/C DP: trypsin/chymotrypsin-digested protein; T/C RP: trypsin/chymotrypsin-resistant protein.

Protein digestibility is a key attribute of protein quality from a nutrition standpoint. Here, we determined the digestibility of individual proteins within the CSMPI proteome through LC-MS/MS profiling of various protein fractions obtained from post-treated CSMPI after digestion using the Megazyme protein digestibility assay kit. The schematic workflow is shown in Figure 5A. Briefly, the post-treated CSMPI was digested sequentially using pepsin and trypsin/chymotrypsin, to mimic the human gastric and intestinal digestive system. The digested peptides and undigested protein fractions were collected after each enzymatic process for LC-MS/MS analysis. Undigested protein fractions were first subjected to urea solubilisation followed by trypsin (sequence grade) digestion to enable bottom-up proteomics analysis. Based on the results, only five proteins were successfully digested with pepsin and 70 proteins with trypsin/chymotrypsin digestion. At the same time, 156 proteins and 43 proteins were identified to be somewhat resistant to pepsin and trypsin/chymotrypsin digestion, respectively.

Next, hierarchical clustering analysis was performed to study the digestibility trend of CSMPI from the gastric and intestinal phases of digestion. Based on the abundance value of each protein in the four fractions, 10 clusters were generated (Figure S3) and none of the proteins were found to be fully digested in the gastric phase. Nonetheless, proteins from Clusters 1 and 4 were efficiently digested in the intestinal phase using the in vitro digestibility kit (Figure 5C). The presence of proteins in the trypsin/chymotrypsin-resistant fraction would suggest that the protein has poor digestibility, and this was observed in proteins from Clusters 3, 7, 10 and 11. In particular, Cluster 7 contains the seed storage proteins (Legumin A and Legumin B), the most abundant proteins in the post-treated CSMPI (Figure 5D). On the other hand, proteins from Clusters 10 and 11 were not digested by the in vitro digestibility kit.

In addition to having ultra-low gossypol content in CSMPI, another aspect of food safety is identifying potential allergenic proteins in CSMPI, which can impede their use as a new source of alternative proteins for human consumption. The top five most abundant proteins in CSMPI were screened for potential allergen features as they can have a profound impact if found to be allergenic. An in silico analysis was performed using the AllergenOnline allergen prediction algorithm (http://www.allergenonline.com/, accessed on 23 June 2022) and the finding was cross validated with AllerCatPro 2.0 (https://allercatpro.bii.a-star.edu.sg/, accessed on 23 June 2022) (Tables S1 and S2). The CSM proteins were associated with allergens from numerous protein sources, but only those that the FDA lists as major allergens were evaluated, and the results are summarized in Figure 5B. Among the top five most abundant proteins, Legumin B (G3M4J0), vicilin A (G3M3J4), and vicilin C72 (A0A1U8LLA0) were found to exhibit strong potential for allergenicity due to matches with tree nut allergens, as well as possessing multiple gluten-like Q-repeats. However, the AllerCatPro analysis did not find any epidemiology studies that confirmed their allergenicity. Therefore, further assessment was conducted on these identified proteins through a database search against the Celiac Database (CD) (accessed on 27 June 2022). None of the top five proteins had more than a 45% identity match over at least half of the FASTA-aligned CD proteins, indicating the unlikeliness of CSMPI in causing coeliac disease.

## 3. Discussion

### 3.1. The Developed Protein Extraction Method and Gossypol Treatment Improve Protein Functionalities

Cottonseed meal is used as livestock feed at present, particularly for ruminants, as they have a higher tolerance against free gossypol toxicity [24]. However, the FDA has mandated a much lower free gossypol concentration (not exceeding 450 ppm) in cottonseed-derived food products for human consumption. We developed an alkaline protein solubilisation process with acidified organic-solvent-based gossypol removal for CSM protein extraction to meet this requirement. A subsequent evaluation was conducted using a proteomics approach to study CSMPIs and their potential as a new source of alternative proteins for human consumption. Before the protein extraction, we sieved the hull and fibre from the CSM to improve the effectiveness of the protein recovery. Pojić et al. highlighted that dehulled meals contain higher protein and lower fibre content, while unhulled meals require an additional fractionation step to achieve high protein recovery [25]. Various acid and alkaline protein extraction protocols have been reported on various plant sources [26]. We adopted the alkaline extraction strategy as this method generally achieves better yields than acidic extraction. In addition, the alkalinity helps to break the cell wall and increases protein solubility [27]. Our improved alkaline extraction method successfully achieved a high protein-recovery yield with excellent purity while preventing protein degradation. Multiple washing steps using acidified ethanol were utilised to remove both free and bound gossypol. Before the gossypol removal treatment, the CSMPI still had a significant amount of free and total gossypol, despite already being significantly lower compared to the unprocessed CSM reported earlier by different research groups [9]. On average, the CSM has been reported to contain around 11,800 ppm TG and 8950 ppm FG [9]. For the post-treated CSMPI, we recorded an ultra-low gossypol level of 4.8 ppm FG and 147.2 ppm TG, significantly lower than the pre-treated CSMPI. In terms of gossypol toxicity, a previous study identified an inhibitory effect on spermatogenesis when consuming free gossypol at a dosage of 0.1 mg/kg body weight [28]. Our post-treated CSMPI had a free gossypol dosage of 0.004 mg/kg body weight, after taking into consideration the daily protein intake requirement of 60 g for a 75 kg adult male. As such, the post-treated CSMPI should be considered generally safe for human consumption. Nevertheless, the clinical impacts and side effects on human health of long-term low-dose gossypol intake remains to be evaluated. We also kept FG removal treatment temperature at no more than 50 °C to maintain protein integrity, as demonstrated by the SDS-PAGE profile of the CSMPI. Furthermore, this treatment temperature is lower than previously reported [19], and will helps toward lowering the cost of production.

The functional properties of the post-treated CSMPI were characterised to verify their suitability for food application. WAC is critical as it impacts food products’ appearance and mouthfeel characteristics [29]. The post-treated CSMPI had a WAC of 3.27 ± 0.03 g/g. This result is higher than the data reported by Ma et al. on various cottonseed protein isolates, which range from 1.4 to 2.9 g/g [30]. The WAC data presented are also complementary to the water solubility figure, which was approximately 47.5%. The solubility provided an important insight into whether the post-treated CSMPI is suitable for food applications. The result also showed that our proposed method effectively removes gossypol and does not affect the protein quality. On the other hand, the OAC, emulsion and foaming properties were generally lower in our post-treated CSMPI compared to the cottonseed protein isolate from Ma et al. [30]. Next, the functional properties of the post-treated CSMPI were benchmarked against pea protein isolate. The results indicated that post-treated CSMPI has a better OAC and water solubility performance. A high OAC is essential in various food processes such as baking and in formulating products such as salad dressing. This allows for better retention of fat flavours, reduces rancidity development and provides a soft mouthfeel outcome [29,31]. CSMPI may be used as a plant-based protein (alternative protein) ingredient for such food applications.

Based on these results, we have developed a proof-of-concept, time-efficient and scalable process to obtain ultra-low-gossypol-content CSMPI. The current process will require further optimization for scaled-up validation, food-grade production, and automation process design before being translatable to pilot-scale production. In addition, an environmentally friendly approach with life-cycle analysis, a zero-waste strategy, and techno-economic evaluation for yield, recovery, and purity is needed. Minimal use or the recycling of chemicals and organic solvents would be ideal for the gossypol removal process.

### 3.2. More Low-Abundance Proteins Identified in Post-Treated CSMPI

Next, SDS-PAGE analysis was performed to determine whether the proteins had been degraded after the gossypol removal steps. We observed that the protein’s integrity was maintained, and the purity improved, though with a small amount of protein loss. The minor protein loss is expected as the washing step involves a small amount of water, in which specific proteins are soluble. Finally, we checked and compared the pre-treated and post-treated CSMPI protein profiles. The number of proteins identified was 178 and 199 proteins, respectively. The increase in protein identification after gossypol removal treatment can be attributed to the improved protein purity after the removal of gossypol and other solvent-soluble contaminants. In particular, the release of bound gossypol from lysine and/or arginine after our gossypol removal treatment would likely improve the protein digestion efficiency during proteomics sample preparation, leading to more protein identification.

Furthermore, the number of proteins identified in this study was much higher than previously reported for glanded cottonseed protein [32]. The data indicated that our protein extraction strategy, coupled with high-resolution LC-MS/MS analysis, improved the identification of low-abundance proteins. Overall, we observed that the gossypol removal treatment facilitated more protein identification in the range of 25–50 kDa and in the pI range of 4–9, where most cottonseed meal proteins were identified. This further illustrates the efficiency of our gossypol removal treatment in improving the detection sensitivity of the low-protein post-treated CSMPI during LC-MS/MS analysis.

### 3.3. Higher Nutritional Content in Post-Treated CSMPI

Based on the proteomics dataset, 95.96% of the post-treated CSMPI proteome consists of seed storage proteins. These proteins are high in essential amino acids such as arginine, leucine and valine, with similar observations in other reports [33,34]. Furthermore, the gossypol removal treatment enhanced the CSMPI’s protein purity from 71% to 92.6%, leading to higher nutritional content per gram of powder. Branched-chain amino acids (BCAAs) such as leucine and valine have been shown to promote muscle protein synthesis by enhancing translational activity through the mTOR signalling pathway [35]. This has led to the application of BCAAs toward sports wellness [36] and also in the management of sarcopenia among the elderly [37]. Additionally, arginine also contributes to skeletal muscle development through mTOR signalling and enhanced vasodilation through arginine-derived nitric oxide [38]. Hence, it would be interesting to evaluate the potential food application of post-treated CSMPI towards skeletal muscle maintenance and growth. We also explored the taste profile of CSMPI peptides and amino acid content via in silico analysis. The top three proteins analysed already consisted of more than 70% of all the proteins, suggesting that the taste exhibited by these proteins would be the dominant taste for CSMPI. The proteins were predicted to have a bitter taste, combined with umami and sweet. The bitter taste of protein hydrolysates has been connected to peptides comprising hydrophobic amino acids previously concealed within the protein structure but exposed after hydrolysis [39]. To transform CSMPI for human consumption, we suggest mixing the proteins with different taste enhancers to ensure the food is acceptable to the public. However, a more in-depth analysis has to be confirmed on the matter.

### 3.4. CSMPI Digestibility Profile through LC-MS/MS Analysis and Identification of Potential Allergen through In Silico Prediction Analysis

The results obtained from the in vitro protein digestion assay using different enzymes mimic the protein digestibility in a human digestive system. Generally, three different phases of protein digestion can be distinguished throughout the gastrointestinal tract [40]. The pepsin digestion requires a low pH condition as pepsin is primarily produced by gastric chief cells in the stomach. However, protein digestion that happens in the stomach is less important compared to the digestion that takes place in the intestinal phase [41]. The digestibility result showed that more proteins were digested by trypsin/chymotrypsin, which is synthesised by the pancreas. The intention of digesting CSMPI is to understand how the extracted proteins will behave when consumed and digested in the human gastrointestinal tract. This method should allows a more comprehensive understanding of digestibility and allergenicity in dietary proteins compared to the traditional pepsin resistance test as both the digested and undigested protein fractions are profiled [42]. Hierarchical clustering analysis of the in vitro digestibility proteomics data identified seven proteins (Clusters 10 and 11) completely resistant to digestion. Additionally, 28 proteins (Clusters 3 and 7), which include the most abundant proteins (Legumin A and B) in our dataset, were incompletely digested. Other than lowering the protein nutritional value, proteins resistant to gastrointestinal digestion may promote food allergy due to possible protein epitopes for IgE binding [43,44,45]. Hence, the ability to study the digestibility profile of each protein within the CSMPI enables a guided approach towards applying additional food processing such as protein hydrolysis, protein cross-linking and/or fermentation, to modulate and enhance the digestibility and nutritional quality of the proteins [46].

CSMPI has not been widely used in food products for human consumption, and the identification of putative allergens in CSMPI will provide invaluable insight into generating and regulating nutritive CSMPI-based food products. We performed an in silico analysis to identify potential allergens from the top five most abundant proteins in CSMPI. The search criteria and filtering process were conducted according to the Food and Agricultural Organization (FAO) and World Health Organization (WHO) panels’ guidelines that were established in 2001 [47]. However, the guidelines were considered overly conservative and ought to be reviewed after over two decades [48]. Therefore, the data were validated using the AllerCatPro 2.0 program due to its superior accuracy in allergen prediction and its ability to provide comprehensive allergen profiling [49,50]. The analysis revealed that three of the five most abundant proteins had features of allergenicity and could be a cause of concern for certain demographics, particularly people with a tree nut allergy or gluten intolerance. Among the three proteins, vicilin A and vicilin C72 were fully digested in our digestibility study; a previous study found that well-digested allergens reduced IgE binding [51]. This indicates that the probability of vicilins being allergenic is further diminished. Furthermore, the occurrence of allergic reactions from cottonseed protein consumption remains rare, despite several recorded incidents [52,53]. In addition, the prevalence of coeliac disease is estimated to be 1.4% of the world population, predominately in European and Oceanic countries [54]. A recent review suggests that IgE cross-reactivity between food species remains low [55], so the cross-reactivity between tree nut allergenic proteins and CSMPI should be negligible. On the other hand, poorly or non-digested legumin A and 2S sulphur-rich seed storage protein 2-like, were determined to have weak evidence of allergenicity by the AllerCatPro software. Overall, we have demonstrated that studying the digestibility profile of the CSMPI proteome, in conjunction with an in silico allergenic prediction analysis, facilitated a comprehensive evaluation of CSMPI’s allergenic potential.

## 4. Materials and Methods

Unless otherwise specified, all chemicals and kits described in the article were obtained from Sigma-Aldrich (St. Louis, MA, USA).

### 4.1. Extraction of Protein Isolate from Cottonseed Meal

Cottonseed (*Gossypium* spp.) meal (CSM) was obtained from Planters Cotton Oil Mill, Inc. (Pine Bluff, AR, USA) The meal was first ground using a benchtop microfine grinder (IKA, Staufen, Germany) and subsequently filtered through a 0.5 mm sieve to obtain a fine CSM powder. A total of 12 g of the CSM was weighed, and 30 mM of sodium hydroxide (NaOH) solution was introduced to the CSM at a ratio of 1:8 (volume:volume). The pH was tested and found to be around 9.5–10.5. The sample was sonicated for 5 min and then stirred using a magnetic stirrer for 60 min at room temperature.

Then, the CSM was centrifuged at 7000× *g* for 15 min, and the supernatant was transferred to a new tube. The pellet was then re-suspended in 30 mM NaOH at a ratio of 1:4 (volume:volume) and stirred for another 30 min. The sample was then centrifuged at 7000× *g* for 15 min, and the supernatant was collected and combined with the previous supernatant. Next, the pH of the supernatant was adjusted to pH 4 to precipitate the proteins. The sample was then incubated on ice for 60 min. Next, the CSM was centrifuged at 7000× *g* for 15 min, and the protein pellet was harvested. Later, the pH of the pellet was adjusted to pH 7.5 using NaOH. The protein isolate was then freeze-dried, and the dry weight was measured. The product yield (Equations (1) and (2)) and protein purity (Equation (3)) of the CSMPI were determined using the Bradford method [56]. The protein isolates (CSMPI) were then weighed and kept at −80 °C until subsequent analysis.

(1) $$\mathrm{Product}\;\mathrm{Yield}\;\left(\text{pre-treat CSMPI}\right)=\frac{\;\mathrm{Pre}\text{-}\mathrm{Treat}\;\mathrm{CSMPI}\;\mathrm{concentration}\;\left(\mathrm{Bradford}\;\mathrm{assay}\right)\;}{\mathrm{Raw}\;\mathrm{CSM}\;\mathrm{weight}}\times 100\%$$

(2) $$\mathrm{Product}\;\mathrm{Yield}\;\left(\mathrm{post}\text{-}\mathrm{treated}\;\mathrm{CSMPI}\right)=\frac{\;\mathrm{Post}\text{-}\mathrm{Treated}\;\mathrm{CSMPI}\;\mathrm{concentration}\;\left(\mathrm{Bradford}\;\mathrm{assay}\right)\;}{\mathrm{Pre}\text{-}\mathrm{treat}\;\mathrm{CSMPI}\;\mathrm{weight}}\times 100\%$$

(3) $$\mathrm{Protein}\;\mathrm{purity}=\frac{\;\mathrm{Pre}/\mathrm{Post}\text{-}\mathrm{Treated}\;\mathrm{CSMPI}\;\mathrm{concentration}\;\left(\mathrm{Bradford}\;\mathrm{assay}\right)\;}{\mathrm{Pre}/\mathrm{Post}\text{-}\mathrm{treated}\;\mathrm{CSMPI}\;\mathrm{weight}}\times 100\%$$

### 4.2. Gossypol Removal Treatment

50 mg of the cottonseed meal protein isolate (CSMPI) was weighed. The CSMPI was then introduced with 0.34 M phosphoric acid in 95% ethanol at a ratio of 1:10 (weight:volume) and incubated under vigorous shaking for 60 min at room temperature. The sample was then centrifuged at 14,000× *g* for 15 min at 4 °C. The supernatant containing the free gossypol was removed. The pellet was re-suspended in 0.34 M phosphoric acid in 95% ethanol at a ratio of 1:5 (weight:volume), and then, incubated under vigorous shaking for 90 min at 50 °C. The sample was then centrifuged at 14,000× *g* for 15 min at 4 °C, and the supernatant was removed. The pellet was air-dried and kept at −80 °C for further analysis.

### 4.3. Extraction of Free and Total Gossypol from Pre-Treated and Post-Treated CSMPI

The CSMPI was mixed with a 1:10 ratio (weight:volume) of 95% ethanol for free gossypol extraction for 15 min under vigorous shaking. The sample was then centrifuged at 14,000× *g* for 15 min at 4 °C, and the supernatant was transferred to a new tube and air-dried.

For total gossypol extraction, the complexing reagent to remove total gossypol was prepared by mixing 4 mL of 3-amino-1-propanol with 20 mL of glacial acetic acid, followed by diluting to 200 mL with N, N dimethylformamide (DMF). The sample was then introduced with complexing agent at a ratio of 1:8 (weight: volume) and shaken vigorously at 90 °C for 30 min. The sample was then cooled down and centrifuged at 14,000× *g* for 15 min at 4 °C to separate the supernatant. This step was repeated once more, and the supernatant was combined and air-dried. Finally, the pellets containing the treated protein isolates were freeze-dried and kept at −80 °C.

### 4.4. Detection of Gossypol Level Using High-Performance Liquid Chromatography (HPLC)

The air-dried free and total gossypols were dissolved in 70% acetone and analysed using the Dionex Ultimate^TM^ 3000 UHPLC Systems (Thermo Fisher Scientific, Waltham, MA, USA). The analysis method of Conceicao et al. was performed with minor modifications [57]. Gradient elution was performed using a mobile phase beginning with 40% aqueous (TFA 0.1%, *v*/*v*) and 60% organic (100% methanol) solvent on a KINETEX^®^ EVO C18 column (150 × 3.0 mm, 2.6 μm) (Phenomenex, Torrance, CA, USA) at 35 °C. A 50 µL sample was injected at a flow rate of 600 µL/min for each run, with a total run time of 16.5 min (including column re-equilibration) (Table 2). Signal detection was observed at 254 nm. Data acquisition and integration were performed using Chromeleon^TM^ Chromatography Data System (CDS) Software, Version 7.2 (Thermo Fisher Scientific, MA, USA).

### 4.5. Water Absorption Capacity (WAC), Oil Absorption Capacity (OAC) and Water Solubility

The WAC and OAC were determined in the post-treated CSMPI using the method developed by Kraithong et al. with minor modifications [58]. The CSMPI consisting of 100 mg were weighed in a microcentrifuge tube before being introduced to 1 mL of water. The mixture was then vortexed for 1 min and incubated for 30 min. Next, the mixture was centrifuged at 1400× *g* for 30 min at room temperature. The supernatant was then discarded, and excess fluid in the tube was drained for 10 min. The tube was then weighed again to identify the amount of water retained by the sample. The same method was performed for OAC determination, with the water being replaced with oil. The calculation for WAC and OAC (Equation (4)) is as follows:

(4) $$\mathrm{WAC}/\mathrm{OAC}\;(\frac{\mathrm{mg}}{\mathrm{mg}})=\frac{\mathrm{weight}\;\mathrm{of}\;\mathrm{wet}\;\mathrm{sample}\;\left(\mathrm{mg}\right)}{\mathrm{dry}\;\mathrm{weight}\;\mathrm{of}\;\mathrm{sample}\;\left(\mathrm{mg}\right)}$$

The water solubility for CSMPI was calculated using the method reported by Kraithong et al. with minor modifications [58]. A total of 100 mg of post-treated CSMPI was mixed into 1 mL of distilled water and vortexed for 60 s. The mixture was heated at 30 °C for 30 min and centrifuged at 1500× *g* for 10 min. The supernatant was then collected and transferred to a new weighed tube and dried. The dried sediments were then weighed. The water solubility (Equation (5)) of the CSMPI was calculated using the following formula.

(5) $$\mathrm{Water}\;\mathrm{solubility}\;\left(\%\right)=\frac{\mathrm{weight}\;\mathrm{of}\;\mathrm{the}\;\mathrm{dried}\;\mathrm{supernatant}\;\left(\mathrm{mg}\right)}{\mathrm{dry}\;\mathrm{weight}\;\mathrm{of}\;\mathrm{sample}\;\left(\mathrm{mg}\right)}\times 100$$

### 4.6. Emulsifying Activity Index (EAI) and Emulsion Stability Index (ESI) Measurement

The EAI measurement was based on Cameron et al. [59] with slight modifications. Briefly, 300 μL of vegetable oil was added to 900 μL of protein solution (1%) and homogenised using the Diax 900 Homogeniser (Heidolph Instruments, Germany) for 1 min. A total of 4 μL of the emulsion was then added to 1 mL of 0.1% SDS and mixed well. The absorbance was read at 500nm, using the EPOCH2 microplate spectrophotometer (Biotek Instruments, Winooski, VT, USA). The EAI (Equation (6)) was calculated as follows:

(6) $$\mathrm{EAI}\;(\frac{m^{2}}{g})=\frac{2T}{c\left(1-\Phi \right)}$$

where T = turbidity (2.303) × (dilution factor (250) × absorbance ÷ pathlength (0.0056 m), c = protein concentration (g/m^3^) and Φ = oil volume fraction (0.25). The calculation of ESI (Equation (7)) was based on Xue et al. [60] using the following formula:

(7) $$\mathrm{ESI}\;(\min)=\frac{A_{0}}{{(A}_{0}{-A}_{1})}\;\times \;∆T$$

where A_0_ = absorbance at zero minutes, and A_1_ = absorbance after 10 min.

### 4.7. Foaming Capacity and Stability Measurement

The foaming capacity (FC) and foaming stability (FS) measurement were adapted from Chao and Aluko [61] with slight modifications. Briefly, 1 mL of protein solution (1%) was added to 3 mL of distilled water and homogenised using the Diax 900 Homogeniser (Heidolph, Germany) for 1 min. The FC (Equation (8)) was calculated using the following equation:

(8) $$\mathrm{FC}\;\left(\%\right)=\frac{V_{1}}{V_{0}}\times 100$$

where V_0_ is the total volume of the protein solution before homogenisation and V_1_ is the total volume of the protein solution after homogenisation. The FS (Equation (9)) was calculated using the following equation:

(9) $$\mathrm{FS}\;\left(\%\right)=\frac{V_{30}}{V_{1}}\times 100$$

where V_30_ is the total volume of the protein solution 30 min after homogenisation.

### 4.8. Sodium Dodecyl Sulphate–Polyacrylamide Gel Electrophoresis (SDS-PAGE)

A total of 40 µg of the pre-treated and post-treated CSMPI was added to 2× Laemmli buffer (Bio-Rad, Hercules, CA, USA) with 50 mM dithiothreitol (DTT) and heated at 95 °C for 5 min; then, they were loaded into a 12% pre-cast Mini-PROTEAN^®^ TGX™ polyacrylamide gel (Bio-Rad, CA, USA) for protein separation. The electrophoresis was set at 200 V, and the gel was run for 35 min. Protein bands were visualised using Coomassie Blue stain, and the gel image was captured using the GE Image Scanner III (Ge Healthcare, Chicago, IL, USA).

### 4.9. Amino Acid Analysis

A total of 50 mg of post-treated CSMPI was hydrolysed with 1 mL of 6 M HCL at 115 °C for 24 h and dried down under a stream of nitrogen gas. A total of 40 mg of dried amino acid was redissolved in 20 mL of de-ionised water. Amino acid derivatization was performed using the AccQ-Tag Ultra Derivatization Kit (Waters Corp, Milford, MA, USA), according to manufacturer’s protocol [62]. Briefly, 10 μL of the reconstituted sample was added to 70 μL of AccQ-Tag Ultra Borate Buffer reagent and 20 μL of AccQ-Tag Ultra reagent. The mixture was then vortexed for 10 s and heated for 10 min at 55 °C. Amino acid standards at concentrations ranging from 1.6 to 500 μM were also prepared in a similar manner to that described above. In addition, a blank reference sample was prepared using 80 μL of AccQ-Tag Ultra Borate Buffer reagent and 20 μL of AccQ-Tag Ultra reagent. The derivatized samples were analysed in triplicate using the UPLC system (Acquity, Waters Corp, USA) equipped with a UV detector set at a wavelength of 260 nm (tuneable ultraviolet (TUV) optical detector, Waters Corp). Separation of the amino acids was achieved using a reversed-phase UPLC column (AccQ-Tag Ultra C18 column, 2.1 × 100 mm, 1.7 μm, Waters Corp) at a flow rate of 0.7 mL/min. The mobile phase system comprised A: a 1-in-10 dilution of AccQ-Tag Ultra Eluent A concentrate with de-ionised water, and B: 98% (*v*/*v*) acetonitrile (ACN) with 2% formic acid (FA). The elution gradient was set as follows: 0.1% B (0–0.54 min), 0.1–9.1% B (0.54–5.74 min), 9.1–21.2% B (5.74–7.74 min), 21.2–59.6% B (7.74–8.04 min), 59.6–90% B (8.04–8.05 min, held for 0.59 min) and 90–0.1% B (8.64–8.73 min, held for 0.77 min). The column was kept at a temperature of 60 °C, while all samples and standards were maintained at 4 °C prior to analysis. The raw data were analysed using the TargetLynx software (version 4.2, Waters Corp) to derive the calibration curves and determine the individual amino acid values for each sample. The average values and coefficients of variation were calculated based on the triplicate runs for all standards and samples.

A molar-ratio normalized sequence-based analysis was performed on the top 10 most abundant proteins in post-treated CSMPI to determine their amino acid composition. The amino acid composition calculated from the in silico analysis was similar to that of the experimentally determined composition in the post-treated CSMPI. As such, the percentage of cysteine, methionine, and tryptophan content, which were not well retained after acid hydrolysis, was calculated through this in silico approach.

### 4.10. In Vitro Digestibility Assay

The in vitro digestibility assay was performed using the Megazyme Protein Digestibility Assay Kit (Megazyme Ltd., Wicklow, Ireland) with some modifications. Briefly, a total of 100 mg of CSMPI was initially mixed with 3.8 mL of 0.06 N HCl and incubated for 30 min at 37 °C with continuous stirring at 300 rpm. Later, 200 µL of pepsin solution (1 mg/mL) was introduced to the CSMPI and incubated for 60 min at 37 °C with continuous stirring at 300 rpm. After the pepsin digestion was complete, the pH of the sample was adjusted to 7.4 using Tris buffer. A total of 200 µL of the soluble mixture was transferred to a new tube (Tube A). The remaining sample in the original tube was then introduced to 5 mg/mL of Trypsin/Chymotrypsin solution and incubated for 4 h at 37 °C with continuous stirring at 300 rpm. The sample was heated for 10 min to quench the digestion. Another 200 µL of the soluble mixture was transferred to a new tube (Tube B).

The samples in tube A and tube B were filtered using a Microcon^®^ 10K MWCO filter unit to obtain the digested peptides. Peptide quantitation was performed using the Pierce™ Quantitative Colorimetric Peptide Assay (Thermo Fisher Scientific, MA, USA). Before mass spectrometry analysis, the digested samples were de-salted using ZipTip C18 resin.

The undigested proteins in both tubes were digested using the protocol from Wisniewski et al. [63], with minor modifications. Briefly, the total protein amount in each tube was evaluated using the Bradford method [44] before being reduced using 66.7 mM dithiothreitol (DTT) for 60 min. The samples were then alkylated with 50 mM iodoacetamide (IAA) before being digested using trypsin overnight at 37 °C. The digested samples were desalted using ZipTip C18 resin prior to mass spectrometry analysis. Separate batches of pre-treated and post-treated CSMPI were digested using trypsin without treatment with the Megazyme Protein Digestibility Assay Kit.

### 4.11. LC-MS/MS Analysis

LC-MS/MS analysis was adapted from Sim et al. with some modifications [64]. The peptides were reconstituted in 1% FA with 2% ACN, and three biological replicates were analysed for each condition. A total of 4 µg of peptide sample was injected for each LC-MS/MS analysis, using the TripleTOF 6600+ mass spectrometer (SCIEX) system with the NanoSpray III Ion Source (SCIEX) and coupled with an Eksigent Ekspert nanoLC 425 (NanoLC Ultra 2D, Eksigent, Toronto, Canada). The peptide sample was first concentrated using an ACQUITY UPLC M-Class Symmetry C18 Trap Column (180 µm × 20 mm, 5 µm, 100Å), and then, separated using a ChromXP C18 column (0.3 × 150 mm, 3 µm, 120Å). The mobile phase buffers used were 0.1% FA (A) and 0.1% FA in ACN (B), and a gradient of 3–80% B was applied for 75 min to facilitate peptide separation, at a flow rate of 5 µL/min. A TOF MS1 spectrum (400–1250 *m/z*) was obtained with an accumulation time of 250.1 ms. The 30 most intense precursors with a charge state of 2–5 (intensity > 200) were selected for fragmentation. MSMS spectra were obtained (100–1500 *m/z*) with an accumulation time of 50 ms.

### 4.12. Database Search

PEAKS^®^ Studio XPro (Bioinformatics Solutions, Waterloo, ON, Canada) was used to perform de novo sequencing and database matching. The *Gossypium hirsutum* proteome was retrieved from the UniProt Knowledgebase (UniProtKB) (accessed in September 2021) and was used for protein identification. Carbamidomethylation was set as a fixed modification; deamidation (NQ) and oxidation (M) were set as variable modifications. The maximum missed cleavage was set at 2. The parent mass error tolerance and precursor mass tolerance were set at 10 ppm and 0.6 Da. A false-detection rate (FDR) < 1% and a significance score (−10logP) of >25 for protein were used for protein acceptance. The minimum unique peptide was set at 2. The maximum variable post-translational modification was set at 3. The average local confidence (ALC) was set at >20%.

### 4.13. Bioinformatics Analysis

The protein sequences of the top 10 most abundant proteins in post-treated CSMPI were obtained from the *Gossypium hirsutum* proteome in UniProtKB. The sensory peptides and amino acids of the top 3 most abundant proteins were identified using Biopep-UWM (http://www.uwm.edu.pl/biochemia/index.php/en/biopep accessed on 15 November 2021) [65].

In silico allergen analyses were performed on the top 5 proteins identified using the AllergenOnline allergen prediction algorithm (http://www.allergenonline.com/ accessed on 23 June 2022). A full FASTA 36 search was selected for the sequence search. The predicted allergens were filtered with an E-score lower than 1 × 10e^−7^, PID > 35%, and at least 80 amino acids overlapping (aao) with the input FASTA sequences. Additionally, the filter process continued by shortlisting identical sequences of at least six amino acids with respect to the input FASTA sequence. The analyses were further validated using the AllerCatPro 2.0 web server [50]. Among the shortlisted allergen proteins, only those food allergens which are recognised by the U.S. Food and Drug Administration (https://www.fda.gov/food/food-labeling-nutrition/food-allergies accessed on 27 June 2022) are included in the final report.

The top 5 proteins were also searched against the coeliac disease database provided in AllergenOnline. Potential matches were filtered based on the following parameters: identity matches > 45% over at least half of the FASTA-aligned CD protein, and E score < 1 × 10e^−16^, indicating the potential for causing coeliac disease.

Hierarchical clustering analysis was performed with the ComplexHeatMap package using R-Studio [66].

## 5. Conclusions

In conclusion, we developed a fast and scalable protein extraction process to produce CSMPI with a high product yield (22%) and protein purity (92.6%). In addition, the post-treated CSMPI had an ultra-low gossypol content with only 4.8 ppm of free gossypol and 147.2 ppm of total gossypol, which is well within the US FDA’s allowable limits. Next, the nutritional quality of the post-treated CSMPI was determined through a series of proteomics analyses. Firstly, SDS-PAGE analysis and the proteome profiling of CSMPI indicated that our optimised protein extraction process did not affect the CSMPI content adversely. Furthermore, the gossypol removal treatment improved protein identification, which can be attributed to the improved protein purity. Subsequent amino acid analysis revealed that post-treated CSMPI had high BCAA content. On the other hand, the digestibility profile of the CSMPI proteome was determined and studied in conjunction with in silico allergen prediction analysis. This approach facilitated a deeper understanding of CSMPI’s nutritional quality. Although several putative allergens in CSMPI were identified, further evaluation of the data suggested that the threat of allergenicity in CSMPI remained low. Overall, the present findings indicate that the CSMPI is generally regarded as safe and is a promising source of alternative protein. Nonetheless, the impact of the long-term consumption of CSMPI-based products and the prevalence and severity of CSMPI allergies will require thorough clinical evaluation in future studies.

## Figures and Tables

**Table 1:** Comparison of various functional properties of post-treated CSMPI against pea protein isolate (PPI). All protein isolate solutions were adjusted to pH 7 before functional characterisation. Standard error mean is calculated based on three biological replicates (unpaired *t*-test, * *p* < 0.05, ** *p* < 0.01, *** *p* < 0.001).

	CSMPI	PPI
**Water absorption capacity (WAC) (mg/mg)**	3.27 ± 0.03 ***	4.41 ± 0.09
**Oil absorption capacity (OAC) (mg/mg)**	2.90 ± 0.06 ***	2.26 ± 0.02
**Water solubility (%)**	47.53 ± 3.12 *	37.28 ± 0.04
**Emulsifying activity index (EAI) (m** **^2^** **/g)**	4.15 ± 0.27 **	11.59 ± 1.19
**Emulsion stability index (ESI) (min)**	115.93 ± 7.13	183.60 ± 44.90
**Foaming capacity (FC) (%)**	27.05 ± 1.43	23.70 ± 0.44
**Foaming stability (FS) (%)**	96.13 ± 0.05	96.77 ± 1.70

**Table 2:** Gradient elution for gossypol detection.

Time (min)	Aqueous-0.1% TFA (%)	Organic-Methanol (%)
0	40	60
8	0	100
11	0	100
12.5	40	60
16.5	40	60

## Data Availability

The mass spectrometry proteomics data have been deposited to the ProteomeXchange Consortium via the PRIDE [67] partner repository with the dataset identifier PXD029330. The raw mass spectrometry data for the digestibility analysis can be provided upon request.

## Supplementary Materials

The following are available online at https://www.mdpi.com/article/10.3390/ijms231710105/s1.
