# Supplementary material for: Cottonseed Meal Protein Isolate as a New Source of Alternative Proteins: A Proteomics Perspective
Source: Int J Mol Sci. 2022 Sep 3;23(17):10105. doi: 10.3390/ijms231710105 (PMC9455987; doi:10.3390/ijms231710105)
Supplement: Supplementary file 1 [file ijms-23-10105-s001.zip › Figures S1-S3.pdf]

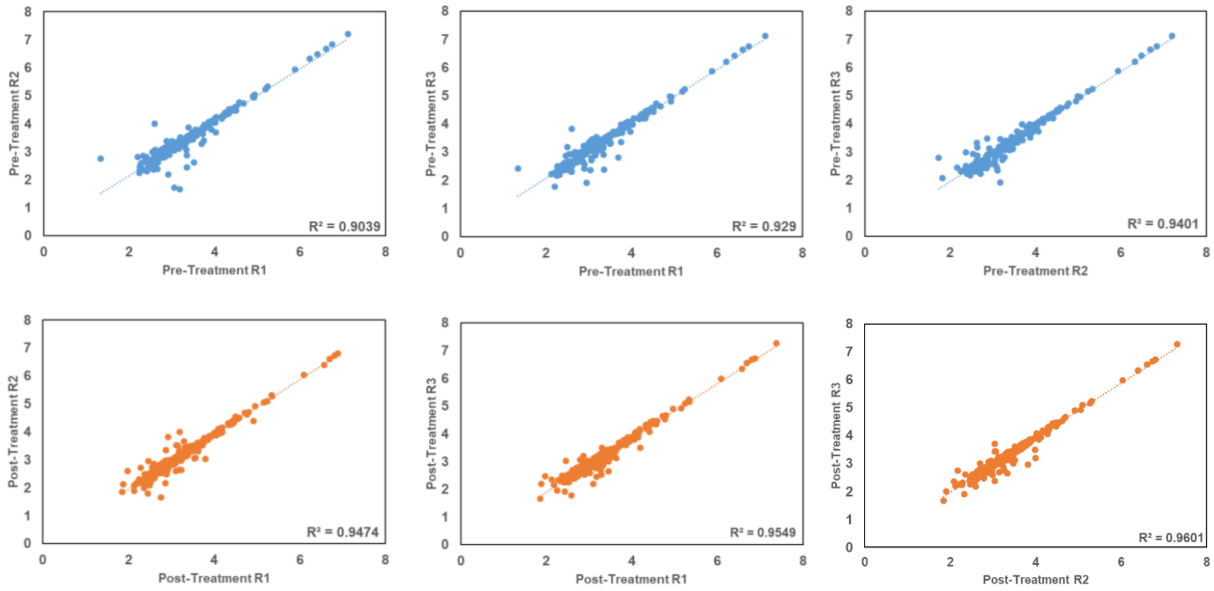

Figure S1: Correlation figures between three biological replicates in the pre-treated CSMPI (top) and post-treated CSMPI (bottom) samples, based on log-transformed data.

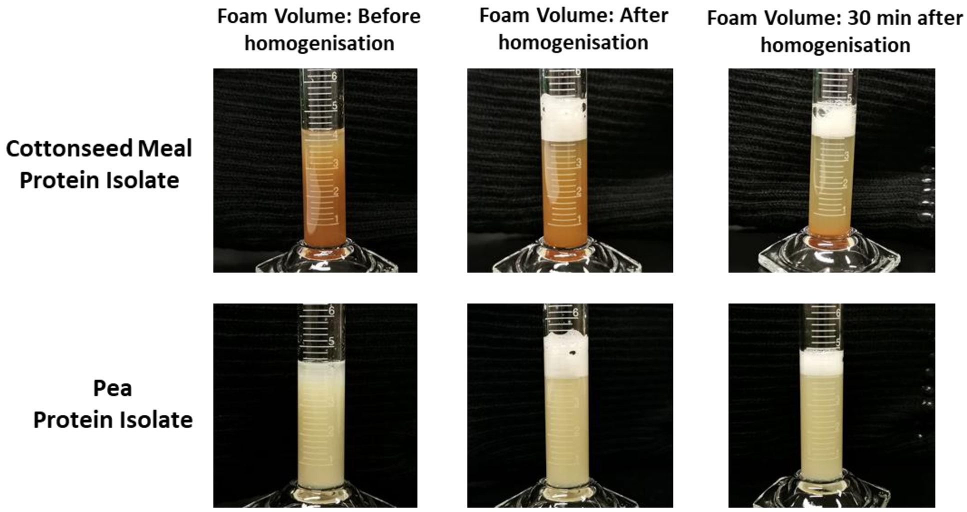

Figure S2: Foaming capacity of cottonseed meal and pea protein isolate. The foam volume was measured before homogenisation, after homogenisation and 30 minutes after homogenisation for foam capacity and stability calculation at 0.25% solution, pH7.

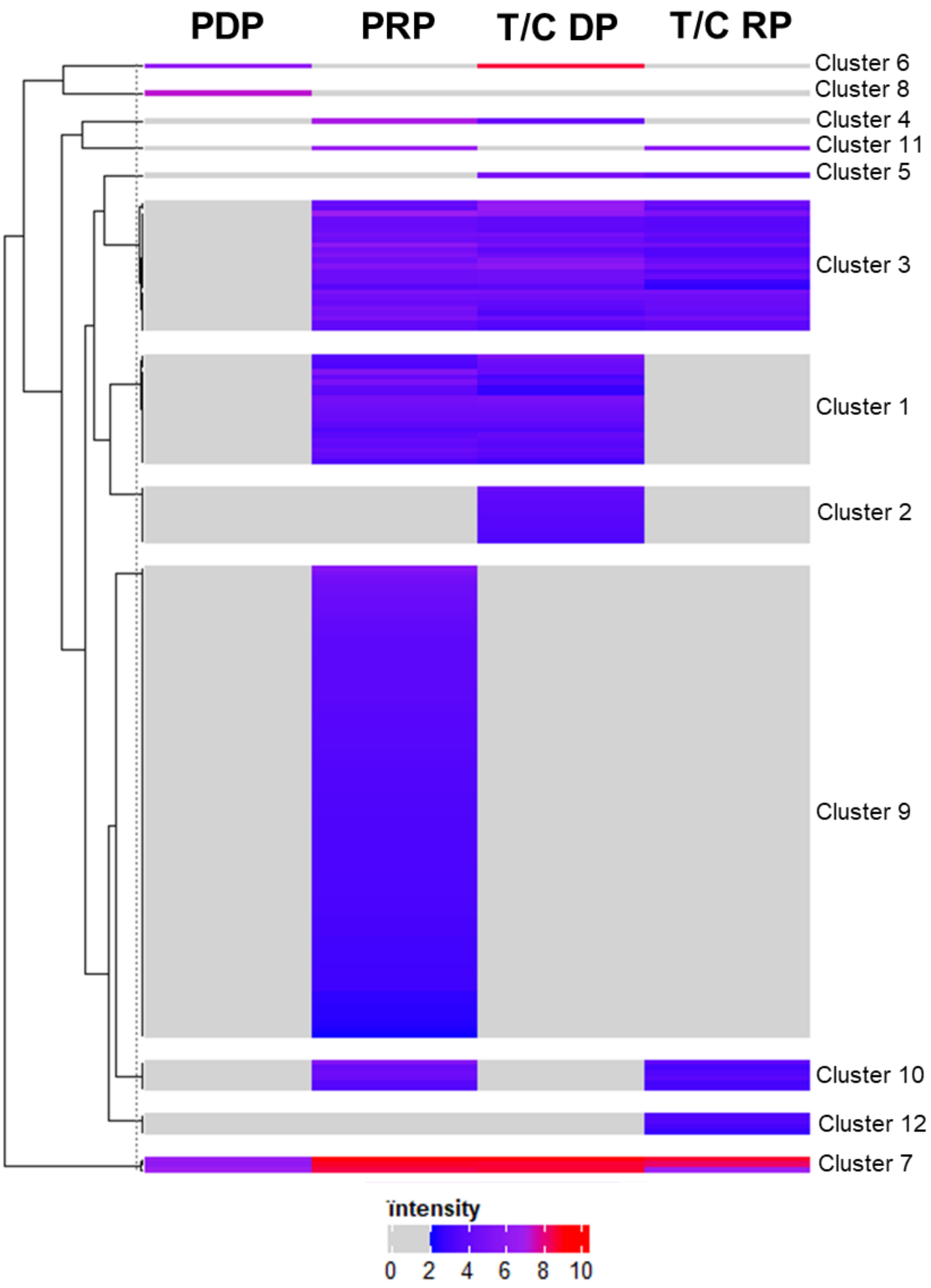

Figure S3: Hierarchical clustering analysis to examine the digestibility profile of individual proteins in the CSMPI.

PDP: *Pepsin-digested protein;* ***PRP:*** *Pepsin-resistant protein;* ***T/C DP:*** *Trpysin/Chymotrpysin-digested protein;* ***T/C RP:*** *Trpysin/Chymotrpysin-resistant protein*
